# Endometrial preparation using gonadotropin-releasing hormone agonist prior to frozen-thawed embryo transfer in women with repeated implantation failure: An RCT

**DOI:** 10.18502/ijrm.v13i5.7150

**Published:** 2020-05-31

**Authors:** Robab Davar, Saeideh Dashti, Marjan Omidi

**Affiliations:** Research and Clinical Center for Infertility, Yazd Reproductive Sciences Institute, Shahid Sadoughi University of Medical Sciences, Yazd, Iran.

**Keywords:** Implantation failure, Gonadotropin-releasing hormone, Embryo transfer, Pregnancy, Implantation.

## Abstract

**Background:**

Preparation of endometrial thickness in frozen-thawed embryo transfer (FET) is extremely important, particularly in repeated implantation failure (RIF) patients.

**Objective:**

This study aimed to investigate the clinical outcomes of FET cycles among RIF women, based on the effects of administering gonadotropin-releasing hormone (GnRH) agonist prior to estrogen-progesterone preparation of the endometrium.

**Materials and Methods:**

In this randomized clinical trial, 67 infertile women who were candidates for FET were divided into two groups: A) case group (n = 34), treated with GnRH agonist prior to endometrial preparation and B) control group (n = 33), which received the routine protocol. (6 mg daily estradiol started from second day) The clinical outcomes) including chemical and clinical pregnancy, in addition to implantation rates, were compared between the two groups.

**Results:**

The results showed no significant differences in women's age (p = 0.558), duration (p = 0.540), type (p = 0.562), and cause of infertility (p = 0.699). Regarding pregnancy and implantation rates, there was a trend toward an increase in the case group; however, differences were not statistically significant.

**Conclusion:**

Although our results showed no significant differences between groups. Because there are trends to better results in case group larger sample size may show significant difference.

## 1. Introduction

Ovarian stimulation commonly increases the number of available embryos for embryo transfer (ET). Despite advances in assisted reproductive technology (ART) over the past four decades, some cycles still fail in terms of achieving live birth following in vitro fertilization (IVF)/ET (1). Embryo cryopreservation can be applied as an important technique in ART programs to avoid multiple ovarian stimulation protocols in infertile couples. Embryo cryopreservation programs enable the creation of surplus embryos following IVF/intracytoplasmic sperm injection (ICSI) to be stored and utilized at a later date in a frozen-thawed ET (FET) cycle (2). Exact synchronization between endometrial maturation and embryo development is an essential factor for successful implantation in FET cycles. Successful FET was reported during a natural cycle following spontaneous ovulation, stimulated cycles (3), and hormone supplement cycles (4). Available hormonal supplement protocols introduced for endometrial preparation differ in terms of drug type, administration route, dosage, and whether or not gonadotropin-releasing hormone (GnRH) analogs are used (5). Recently, in the most common protocols, spontaneous ovulation prior to estradiol and progesterone administration was avoided by pituitary down-regulation with a GnRH agonist (6).

One of the main challenges of ART programs is repeated implantation failure (RIF). Several treatment strategies have been suggested for this problem (7). FET cycles can be timed with ovulation in natural cycles or after endometrial preparation following exogenous hormone therapy (3, 6). Another protocol for controlled endometrial preparation is using the GnRH agonist prior to steroid administration, resulting in ovarian function suppression, subsequently creating synchronization between endometrial and embryo development (8). Several studies showed significant improvements in pregnancy rates for both fresh (9, 10) and frozen cycles (11) following GnRH agonist administration in infertile women with endometriosis or adenomyosis. Specifically, in FET cycles, it was reported that pretreatment with a GnRH agonist in patients with adenomyosis improved endometrial receptivity, as well as pregnancy outcomes (11).

The present study was conducted to investigate the effects of GnRH agonist administration on the implantation rate in the FET cycles of women with RIF, prior to estrogen-progesterone preparation of the endometrium.

## 2. Materials and Methods

### Participants

In the present randomized clinical trial, 67 infertile women with history of idiopathic RIF (at least two implantation failures), undergoing FET cycles in Yazd Reproductive Sciences Institute, Yazd, Iran between August and November 2017 were randomly enrolled in two groups. Randomization was done as computer generated.

The case group (n = 34) received 0.1 mg/day of the GnRH agonist (Variopeptyl, VarianDarou, Iran), subcutaneous, from day 21 of the cycle preceding the actual FET cycle. On the second day of the cycle, the dose of GnRH agonist was reduced to 0.05 mg and 6 mg/day oral estradiol valerate (2 mg, Aburaihan Co., Tehran, Iran) was also started. When the endometrial thickness reached to 7.5 mm, vaginal supplementation of CyclogestⓇ pessaries (Cox Pharmaceuticals, Barnstaple, UK) at 400 mg twice daily was started and the GnRH agonist was also stopped.

**Figure 1:**
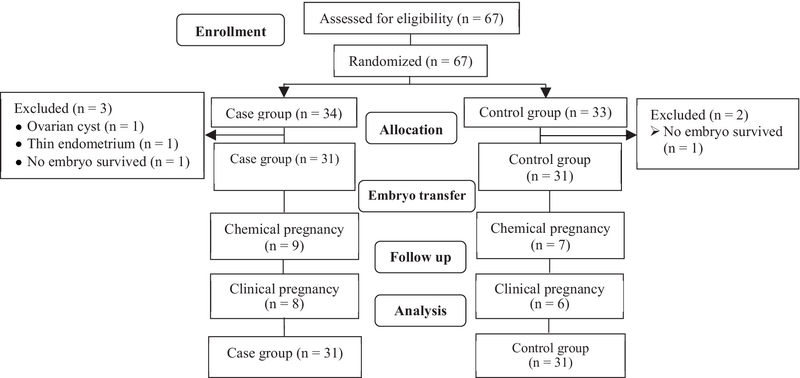
Study design flowchart.

The control group (n = 33), received 6 mg/day oral estradiol valerate (2 mg, Aburaihan Co., Tehran, Iran) from the second day of the cycle without the GnRH agonist. In the two groups, frozen-thawed embryos were transferred on the fourth day of progesterone treatment. All women with endometrial polyp, uterine myoma, and uterine anomaly were excluded from the study (Figure 1).

### Embryo vitrification and thawing

Vitrification and warming procedures were effected using RapidVit${}^{\mathrm{TM}}$ Cleave and RapidWarm${}^{\mathrm{TM}}$ Cleave kits, respectively (Vitrolife Inc., Gothenburg, Sweden), as per manufacturer instructions. The quality of embryos was evaluated 24 h later, and those with less than 50% fragmentation were selected for ET.

### Clinical outcomes 

Chemical pregnancy was defined by serum β hCG > 50 IU/L, 14 days after ET. Clinical pregnancy was approved by the detection of a fetal heartbeat 2 wk after positive β hCG. Implantation rate was defined as the number of gestational sacs per embryos transferred. Moreover, endometrial thickness was observed in sagittal view via sonography on the day that progesterone was started.

### Ethical consideration

The study protocol was approved by the Ethics Committee of Yazd Reproductive Sciences Institute, Shahid Sadoughi University of Medical Sciences, Yazd, Iran (Code: IR.SSU.RSI.Rec.1396.1). Written informed consent was provided by all participants.

### Statistical analysis

Statistical analysis was carried out using the statistical package for the social sciences (SPSS v. 14, Chicago, IL). Both Mann-Whitney U and chi-square tests were performed where appropriate. The level of significance was set at p-value < 0.05.

## 3. Results

In the case group, three cycles were canceled due to ovarian cyst (37 mm), endometrial failure to reach a thickness of at least 7.5 mm, and no embryo survival after thawing procedure. Two cycles were also canceled in the control group because no embryo survived following the thawing procedure.

Finally, the number of cycles remaining was 31 in each group. No participants were lost to follow-up in either group. No significant differences in the demographic characteristics were observed between the two groups (Table I). The two groups were similar in endometrial thickness at the start of progesterone therapy (8.70 ± 1.08 vs. 8.94 ± 1.33, p = 0.521) and in the number and quality of transferred embryo per women (1.90 ± 30 vs. 1.83 ±.52, p = 0.554) (Table I).

As presented in Table II, the ART outcomes in the two groups were not significantly different. Data showed higher chemical and clinical pregnancy rates for the case group than the control group (29% vs. 22.6% and 25.8% vs. 19.4%, respectively); however, differences were not significant. Moreover, implantation rate showed no statistically significant difference between groups; however, there was a trend toward greater implantation rate in the case group (13.55% vs. 10.52%, p = 0.521).

**Table 1:** Comparison of demographic characteristics in two study groups


**Variables**		**Case group (n = 31)**	**Control group (n = 31)**	**p-value**
Age (y)*		32.61 ± 4.40	31.96 ± 4.22	0.558${}^{a}$
Duration of infertility (y*)		6.93 ± 3.94	7.48 ± 2.99	0.540${}^{a}$
Type of infertility **				
	**Primary **	22 (71.0)	24 (77.4)	
	**Secondary**	9 (29.0)	7 (22.6)	<brow>-2</erow> 0.562${}^{b}$
Cause of infertility *				
	**Male factor**	9 (29.0)	13 (41.9)	
	**Poly cystic ovarian syndrome**	5 (16.1)	4 (12.9)	
	**Tubal factor**	3 (9.7)	1 (3.2)	
	**MIX**	9 (29.0)	8 (25.8)	
	**Unexplained**	5 (16.1)	4 (12.9)	
	**Ovarian factor**	0 (0.0)	1 (3.2)	<brow>-6</erow> 0.699${}^{b}$
Endometrial thickness (mm)*		8.70 ± 1.08	8.94 ± 1.33	0.521${}^{b}$
Cycle day*		15.38 ± 2.29	16.38 ± 1.92	0.068${}^{b}$
No. of transferred embryos*		1.90 ± 30	1.83 ± 0.52	0.554${}^{b}$
Embryo quality **				
	**Grade A**	22 (71)	20 (64.5)	
	**Grade B**	7 (22.6)	5 (16.1)	
	**Grade C**	2 (6.5)	6 (19.4)	<brow>-3</erow> 0.297${}^{b}$
* Data presented as Mean ± SD. ** Data presented as n (%) a: Mann-Whitney U test, b: Chi-square						

**Table 2:** ART outcomes for the case group vs. control group


**Variables**	**Case group (n = 31)**	**Control group (n = 31)**	**p-value**
**Chemical pregnancy**	9 (29.0)	7 (22.6)	0.562
**Clinical pregnancy**	8 (25.8)	6 (19.4)	0.544
**Implantation rate**	8/52 (13.55)	6/57 (10.52)	0.521
Data presented as n (%). Analyzed by Chi-square test			

## 4. Discussion

The present study found that pituitary down-regulation prior to frozen ET did not lead to significantly higher chemical and clinical pregnancy rates in women with a history of RIF.

RIF is an important challenge in ART programs for both patients and physicians. An important factor in implantation is synchronization between embryo development and endometrial maturation. One study have evaluated other factors that may affect or improve ART outcome of frozen-thawed embryos (1).

The programmed cycle using a GnRH agonist prior to estrogen and progesterone administration aims to effect pituitary down-regulation, thereby avoiding spontaneous ovulation and cycle cancelation, the primary drawbacks of a natural cycle protocol (12). Our study revealed that the GnRH agonist in women with RIF may improve implantation and pregnancy rates; however, indicated differences showed no level of significance. Hebisha and colleagues showed that daily subcutaneous administration of 0.1 mg GnRH agonist (Triptorelin) prior to estrogen and progesterone (starting from the mid-luteal phase of the previous cycle) increased implantation and pregnancy rates (13). A recent study related to sample size also delivered meaningful results. The implementation of a smaller case group in our study was the reason we did not report significant statistical results. Furthermore, using the GnRH agonist prior to frozen ET in polycystic ovary syndrome, women indicated an improvement in ongoing pregnancy rate (14).

Similarly, Xing and associates found that hormonally controlled endometrial preparation with prior GnRH agonist suppression can be used for patients who had experienced repeated IVF treatment failures, despite having morphologically optimal embryos. This study administered two intramuscular injections of Diphereline (2.5 mg), with a duration time of 28 days between the first and second injection. The comparison showed that clinical outcomes were significantly better with GnRH agonist protocols when compared with endometrial preparation with estrogen and progesterone only protocols in the same patients (15). Generally, the underlying mechanism of a GnRH agonist is not yet clearly understood. Probably It seems that induces pituitary suppression and decreased circulating estrogen, a GnRH agonist may reduce concentrations of peritoneal fluid metalloproteinase tissue inhibitors, down-regulate peritoneal fluid inflammatory proteins, and promote apoptosis and expression of pro-apoptotic proteins (16, 17). Niu and colleagues showed using long-term GnRH agonist prior to IVF/ICSI in infertile women with endometriosis or adenomyosis significantly enhanced the chances of pregnancy in both fresh and frozen cycles (11). In fact, it was found that women with repeated implantation failure have concomitant uterine adenomyosis and a prominent aggregation of macrophages within the superficial endometrial glands, that interrupted with embryo implantation. Tremellen and colleagues showed that pituitary suppression with a GnRH agonist increased implantation rate (18).

In agreement with our result, Dal and colleagues reported no difference in implantation and pregnancy rates with or without the use of a GnRH agonist for pituitary suppression in FET cycles. They administered 3.75 mg Decapeptyl in the mid-luteal phase of the cycle. Their results indicated that endometrial preparation with GnRH agonist pretreatment did not increase the success rate of FET cycles (19). Previously, we found no significant different implantation or pregnancy rates when using exogenous steroids with or without GnRH agonist during FET cycles (20).

## 5. Conclusion

In conclusion, our study found that the use of a GnRH agonist in women with RIF may improve endometrial receptivity and increase pregnancy rate. However, differences were not statistically significant. Therefore, additional large prospective studies are necessary on this topic.

##  Conflict of Interest

The authors declare no conflict of interest.
